# Challenges and Strategies of Informal Caregivers in Medication Management for Older Adults: A Mixed Method Systematic Review

**DOI:** 10.1111/opn.70094

**Published:** 2026-07-11

**Authors:** Daniela Avgustini, Mayra Veronese, Anna Brugnolli, Matteo Danielis, Jessica Longhini

**Affiliations:** ^1^ Azienda Provinciale per i Servizi Sanitari Trento Italy; ^2^ Laboratory of Studies & Evidence‐Based Nursing, Department of Cardiac, Thoracic, Vascular Sciences and Public Health University of Padova Padova Italy; ^3^ Interdepartmental Medical Science Centre University of Trento Trento Italy

**Keywords:** adherence, burden, caregivers, medication, nursing, older adults

## Abstract

**Background:**

As populations age worldwide, older adults increasingly face polypharmacy and medication adherence challenges. Informal caregivers often assume primary responsibility for medication management, yet their experiences remain underexplored beyond dementia care.

**Aim:**

This review aimed to synthesize the experiences of informal caregivers in managing medications for older adults.

**Methods:**

A mixed method systematic review was conducted following the Joanna Briggs Institute (JBI) methodology. Four databases (PubMed, CINAHL, PsycINFO and Scopus) were searched for studies published between January 2015 and January 2025. Eligible studies included qualitative, quantitative or mixed methods research addressing medication management by informal caregivers of older adults (≥ 65 years) living at home. Data were qualitized and integrated through a convergent synthesis approach.

**Results:**

Sixteen studies met the inclusion criteria, comprising 11 qualitative and five cross‐sectional designs across diverse countries. Two integrated findings were identified from the analysis. First, *perceiving and enacting medication management responsibilities*: caregiver involvement often began after acute health events or functional decline and intensified with increased dependency, especially in co‐residing contexts. Their involvement ranged from supervisory support to full responsibility and included obtaining medications, organizing pillboxes, administering doses and monitoring adherence and potential side effects. Medication management was frequently perceived as burdensome, particularly among women, younger caregivers and those managing complex regimens or parenteral therapies. Second, *facing and addressing challenges in medication management*: caregivers reported difficulties with polypharmacy, complex formulations, patient resistance, systemic barriers and limited access to guidance and support from healthcare professionals. Strategies included pill organizers, reminder systems (e.g., alarms or smartphone apps), handwritten medication lists and individualized routines, though many caregivers reported errors and reliance on unverified information sources.

**Conclusions:**

Informal caregivers play a critical yet demanding role in medication management for older adults, often without adequate preparation or support. Their responsibilities span from acquisition to administration and monitoring, placing them at risk of stress, errors and emotional burden. Findings highlight the urgent need for structured interventions providing education, anticipatory guidance and ongoing professional support to strengthen caregiver competence, enhance medication adherence and reduce caregiver burden.

**Implications for Practice:**

Healthcare professionals should systematically assess informal caregivers' readiness, competencies and support needs related to medication management, recognizing them as key partners in care. Tailored education, medication reconciliation, practical training and clear communication should be integrated into routine care, particularly during care transitions. Structured caregiver support may improve medication safety and adherence while reducing caregiver burden and medication‐related errors.

## Summary

**What does this research add to existing knowledge in gerontology?:**

- This review highlights that informal caregivers of older adults (≥ 65 years) living in home and community settings frequently assume substantial responsibility for managing complex medication regimens, ranging from supervisory support to full responsibility depending on the older adult's level of dependency, which may expose them to significant burden and risk of errors.
- It shows that informal caregiver strategies, although creative and adaptive, often emerge without professional guidance and rely on unverified sources, revealing a critical gap in caregivers' education.

**What are the implications of this new knowledge for nursing care for and with older adults?:**

- Nurses are positioned to play a central role in supporting caregivers due to their continuous contact with patients and families across care settings as well as their recognized role in patient education and medication management. Through anticipatory guidance, individualized education and ongoing support, nurses can help caregivers manage medications safely in home and community settings.
- Focusing on nurse‐led interventions is supported by evidence highlighting nurses' contribution to medication safety, care coordination and caregiver empowerment. Strengthening caregiver competence through these interventions can improve adherence, reduce errors, mitigate caregiver distress and prevent the perceived burden.

**How could the findings be used to influence practice, education, research, and policy?:**

- Policymakers should prioritize structured, evidence‐based caregiver support programmes that address medication management as a distinct and critical dimension of informal care.
- Future research and education should focus on co‐designed interventions, integrating caregiver perspectives into training curricula and clinical practice to enhance safe, person‐centred medication management.

## 1 Introduction

Medication management in older adults is increasingly complex, particularly in the context of multimorbidity and polypharmacy (Kurczewska‐Michalak et al. 2021) and often requires the active involvement of informal caregivers (Adeyemi et al. 2025). Ageing of the population further contributes to this growing complexity, as an increasing number of older individuals live with multiple chronic conditions that require multiple medications (Crimmins 2021; World Health Organization 2021).

Polypharmacy, commonly defined as the use of five or more medications, is associated with a higher risk of adverse outcomes and poses significant challenges to the use of safe medications (Pazan and Wehling 2021). Older adults are particularly vulnerable due to age‐related physiological changes, complex treatment regimens, and cognitive decline affecting memory, attention and executive function. These factors can compromise their ability to manage medications independently and adhere to prescribed therapies (Jandu et al. 2024). In addition, normal ageing is often associated with mild cognitive decline, including memory impairment, reduced attention and executive dysfunction, which may further compromise the ability of older adults to manage medications safely and independently (Colita et al. 2024; Randhawa and Varghese 2023). Medication adherence plays a central role in this context, as poor adherence is a main cause of treatment failure (Aremu et al. 2022; Religioni et al. 2025). In nursing practice, assessing adherence, identifying potential barriers and providing individualized education and support are key strategies in the care of older adults, with direct implications for patient safety and clinical outcomes (Sharma et al. 2024). Optimal adherence leads to a range of positive clinical outcomes, including reduced hospitalization rates, fewer disease‐related complications, increased treatment safety and efficacy, better disease management, lower healthcare costs and enhanced quality of life (Religioni et al. 2025).

However, older adults may face several challenges in managing their medications independently due to several reasons (Badawoud et al. 2024; Christopher et al. 2022). These include difficulties in handling polypharmacy, limited awareness of side effects, cognitive issues with understanding, memory and attention, and reduced access to healthcare services (Christopher et al. 2022). To address these issues, informal caregivers frequently assume full or partial responsibility for medication‐related tasks, ranging from preparation to administration and monitoring (Gillespie et al. 2014). Informal caregivers are typically defined as unpaid individuals, often family members, partners or friends, who assist a person with health or functional needs outside formal healthcare services (Shajani and Snell 2023). Informal caregivers play a particularly important role in medication management in home and community settings, where healthcare professionals are not continuously present and responsibility for daily medication administration often falls to relatives or close supporters (Look and Stone 2018, 2019; Pollock et al. 2021). Approximately 80% of caregivers involved in medical tasks report managing medications, including organizing pillboxes, and in some cases, administering intravenous fluids and injections (Reinhard et al. 2012). This task often challenges caregivers and leaves many feeling unprepared to manage intricate medication regimens. In fact, some caregivers report difficulties with specific aspects of medication management, such as ensuring a consistent supply of medications, administering them correctly, making clinical decisions or communicating effectively with healthcare professionals and care recipients (Look and Stone 2019). These difficulties become even harder in some specific diseases, like dementia, where a recent study by Onda et al. (2024), underscores that 38.9% of caregivers experienced burden related to medication assistance, 36.6% reported instances of medication refusal, and 15.5% perceived a mismatch between prescribed treatments and the care recipient's lifestyle when medication schedules or management routines were difficult to integrate into the patient's daily habits or disrupted established routines (Onda et al. 2024).

Despite the central role informal caregivers play in supporting medication management for older adults, their experiences remain insufficiently explored. In particular, few studies address how informal caregivers navigate medication management challenges in everyday care, the challenges they encounter and how these experiences may affect the quality and safety of care provided at home. Existing literature focuses primarily on patients and their informal caregivers with dementia (Aston et al. 2017), and the most recent systematic review regarding only informal caregivers in this context was published 10 years ago (Gillespie et al. 2014). However, recent evidence suggests that medication management challenges also extend beyond dementia populations, including other contexts such as post‐discharge care, where older adults and their caregivers report difficulties in understanding medication instructions, managing changes in therapy and accessing appropriate support (Wood et al. 2026). This highlights the need for an updated and comprehensive synthesis of the literature on the experience of informal caregivers in the medication management of older adults, and this review aims to provide this gap.

## 2 Methods

### 2.1 Aim and Research Questions

The aim of this review is to synthesize the experience of informal caregivers (hereafter, caregivers) in managing medications of older adults.

Specifically, the research questions of the study are: (a) How do informal caregivers perceive and carry out medication management in older adults? (b) What challenges and strategies do informal caregivers report in managing medications in older adults?

### 2.2 Study Design

We conducted a mixed method systematic review following the Joanna Briggs Institute (JBI) methodology with a convergent integrated approach since the review questions can be addressed by both quantitative and qualitative research designs (Stern et al. 2020). A mixed method systematic review was chosen for its methodological rigour in integrating qualitative and quantitative evidence, particularly during the analysis phase. This approach enables the generation of integrated findings relevant to practice, which are interpreted in light of the methodological quality of the included studies, thereby supporting a judgement on the overall reliability of the results (Noyes et al. 2019).

This review was reported according to the Preferred Reporting Items for Systematic Reviews and Meta‐Analysis (PRISMA) guidelines (Page et al. 2021) (File S1).

### 2.3 Search Methods

A comprehensive literature search was conducted on the following electronic databases: PubMed, CINAHL, PsycINFO and Scopus. In addition, the reference lists of all included studies and other relevant articles were manually screened to identify potentially eligible studies not retrieved through the electronic database search. The search strings are reported in File S2.

Inclusion criteria were: (a) design: primary studies employing qualitative, quantitative or mixed methods designs, (b) topic: focused on medication management by informal caregivers of older adults (≥ 65 years old) living at home. For the purpose of this review, informal caregivers were defined as unpaid individuals, including family members, friends or neighbours, who provide care or support to older adults outside formal healthcare services (Oh et al. 2024; Shajani and Snell 2023).

Exclusion criteria were: (a) studies focusing exclusively on formal or paid caregivers; (b) studies not addressing medication management; (c) studies including older adults who were hospitalized or residing in nursing homes or long‐term care facilities; (d) reviews, editorials, conference abstracts and protocols; and (e) studies not available in English.

The search was limited to studies published between January 2015 and January 2025 to provide an updated synthesis of the literature (Gillespie et al. 2014). Earlier studies focusing on medication management among caregivers have been partially synthesized in previous reviews, particularly those focusing on dementia populations. Our review, therefore, prioritized the most recent decade, when medication practices, caregiver roles, and health system contexts have substantially evolved.

### 2.4 Study Selection

All identified records were initially imported into the SR‐Accelerator web tool to remove duplicates. Subsequently, the remaining studies were uploaded to Rayyan, a web‐based platform for systematic review management. Titles and abstracts were independently screened by two reviewers (*D.A*., *J.L*.) according to the predefined inclusion criteria. Full‐text articles of studies deemed potentially eligible were then independently screened by two reviewers (*D.A*., *J.L*.). Discrepancies were solved through discussion, without requiring a third reviewer's opinion.

### 2.5 Quality Appraisal

The methodological quality of all included studies was independently assessed by two reviewers (*D.A*., *J.L*.) using the standardized critical appraisal tools developed by the Joanna Briggs Institute (JBI), appropriate to each study design (Lockwood et al. 2015; Moola et al. 2020).

The JBI checklist for analytical cross‐sectional studies consists of eight appraisal items, whereas the checklist for qualitative research includes 10 items (Moola et al. 2020). Each criterion was rated as ‘+’, ‘−’ or ‘N/A’. The quality assessment was further enhanced by categorizing the methodological quality of studies based on the percentage of criteria satisfied: studies with ≥ 80% of criteria satisfied were rated as high quality, 50%–79% as moderate quality and < 50% as low quality (Heinze 2025; Samadbeik et al. 2024). Any discrepancies between reviewers were resolved through discussion with a third reviewer (*M.V*.).

### 2.6 Data Extraction

Data extraction was independently carried out by two reviewers (*D.A., J.L*.) using an Excel file piloted on two studies.

Extracted information included: study design and data collection methods, inclusion criteria, sampling strategies, sample characteristics, study aims and key findings relevant to the objectives of this review (e.g., caregiver burden, types of medication errors, tools and strategies used). At this stage, no synthesis or interpretation of results was conducted; findings were extracted in their reported form and prepared for subsequent analysis.

### 2.7 Data Analysis

A convergent integrated synthesis approach was adopted, and quantitative data transformation, as recommended by JBI guidance, was performed to obtain data in a mutually compatible format (Stern et al. 2020). In data transformation, quantitative findings were converted into textual descriptions (*qualitizing*) (Stern et al. 2020). Textual descriptions (*qualitized* data) from quantitative studies were merged with qualitative data from qualitative studies in a unified dataset. Then, reviewers conducted repeated, detailed examinations of the data to identify categories based on similarity in meaning and categories were then aggregated into integrated findings (Stern et al. 2020). The process was conducted in all phases independently by two reviewers (*D.A*., *J.L*.), and divergences were discussed with a third author (*M.V*.) until consensus was reached.

## 3 Results

### 3.1 Characteristics of Studies

**FIGURE 1:**
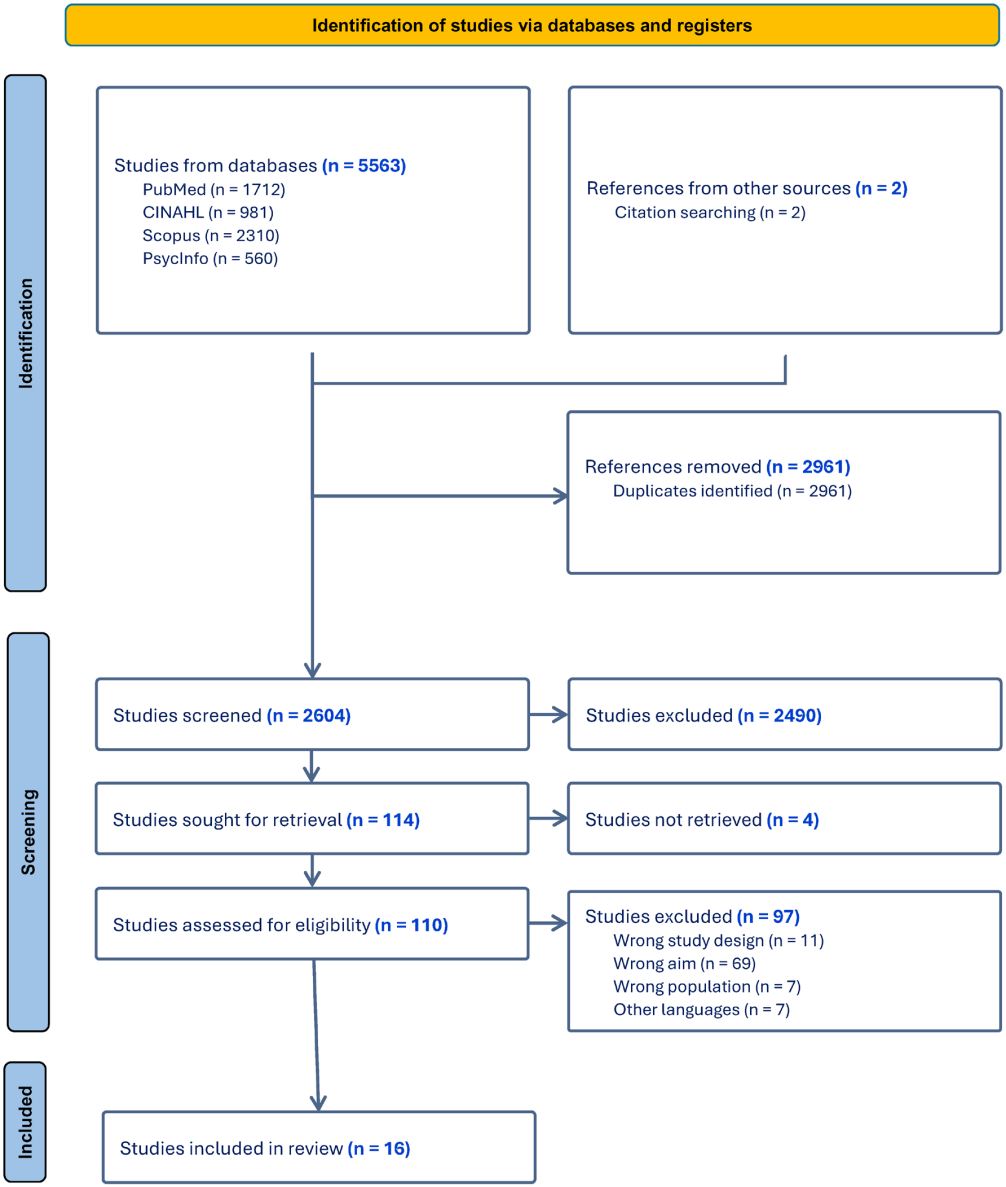
PRISMA 2020 flow diagram of the study.

A total of 5563 records were identified, of which 16 met the inclusion and exclusion criteria (Figure 1, Table 1). Among the included studies, 11 employed qualitative designs (El‐Saifi et al. 2019; Lampert et al. 2020; LaValley et al. 2023; Lawson et al. 2022; Lim et al. 2024; Look and Stone 2018, 2019; O'Conor et al. 2021; O'Conor et al. 2024; O'Quin et al. 2015; Pereira et al. 2022) and five were cross‐sectional studies (Gil‐Hernández et al. 2024; Li and Look 2022; Noureldin and Plake 2017; Onda et al. 2024; Polenick et al. 2020).

**TABLE 1:** Characteristics of the studies included.

Authors, years, country	Study design	Study objective	Data collection method	Inclusion criteria/sampling method	Sample characteristics
El‐Saifi et al. (2019) Australia	Qualitative	To explore factors influencing medication adherence and how they can be addressed	Semi‐structured telephone interviews	/	Recruitment via caregiver support agencies or social media. *N* = 20: Caregiver age: < 65 years (70%), > 65 years (30%) Caregiver gender: 17 females (85%) Care recipient age: 56–92 years
Gil‐Hernández et al. (2024) Spain	Quantitative, cross‐sectional	To investigate factors influencing medication errors by informal caregivers during home care	Online questionnaire	Caregivers: age > 18 years; active caregiving at time of survey or within the past year	Recruitment via invitations sent to all registrants in national caregiver database and caregiver associations. *N* = 685 Caregiver mean age: 51 Caregiver gender: 530 females (87.5%)
Lampert et al. (2020) Germany	Qualitative	To evaluate medication users' experiences in medication administration, perceived causes of issues and individual support needs	Focus groups	Caregivers: age > 18 years; managing ≥ 3 medications, including at least one high‐risk drug	Recruitment through community pharmacies. *N* = 11 Caregiver mean age: 75 (range: 63–76) Caregiver gender: 81% females
LaValley et al. (2023) USA	Qualitative	To understand caregivers' experiences with traditional medication administration strategies, perceptions of new assistive tools, and experiences with new technologies	Semi‐structured interviews (telephone or in‐person)	Caregivers: caring for a loved one > 65 years old—contacted the patient's physician at least once Patients: taking > 1 medication daily	Recruitment via flyers at community events, emails/letters from university databases. *N* = 20 Caregiver mean age: 56 Caregiver gender: 16 females (80%) Care recipient mean age: 81
Lawson et al. (2022) UK	Qualitative	To enhance understanding of the burden on family caregivers managing complex medication regimens for older adults with multi‐morbidity and propose practice/policy improvements	Interviews face‐to‐face	/	Recruitment through professional networks and national television advertisements. *N* = 16 Caregiver; gender: 11 females (68%)
Li and Look (2022) USA	Quantitative, cross‐sectional	To examine the association between caregiver characteristics and difficulties in medication management tasks	Questionnaire	Patients: received help in the past month for self‐care or household activities Caregivers: Unpaid; Assisted with self‐care, household management, transport (last month) or financial/medical management (last year)	Participants identified through the NHATS study. One caregiver randomly selected per patient. *N* = 620 Caregiver age: > 60 years (56.7%) Caregiver gender: 432 females (69.8%)
Lim et al. (2024) UK	Qualitative	To understand how people with dementia and their informal caregivers manage medications at home, identifying opportunities for improvement	Semi‐structured interviews (previous study data)	Patients: Alzheimer's or mixed dementia diagnosis, MoCA score > 9, prescribed at least one chronic medication, living at home, receiving caregiver assistance, ability to consent	Recruitment from the Berkshire Healthcare NHS Foundation Trust Research Interested List. *N* = 14
Look and Stone (2018) USA	Qualitative	To explore how informal caregivers manage medications for older adults, identifying tasks and facilitating tools/strategies	Focus groups	Caregivers: assisting with medication management for an elderly friend/relative > 65 years old	Recruitment via letters to caregiving programme participants, newsletter ads, county ageing department referrals and caregiver conferences. *N* = 29 Caregiver mean age: 67 (range: 42–85) Caregiver gender: 24 females (82%) Care recipient mean age: 83
Look and Stone (2019) USA	Qualitative	To identify contextual factors influencing medication management by rural informal caregivers of older adults	Focus groups	Caregivers: assisting with medication management for an elderly friend/relative > 65 years old	(same as above)
Noureldin and Plake (2017) USA	Quantitative, cross‐sectional	To explore caregiver, care recipient and caregiving‐related factors associated with caregiver involvement in medication ordering, tracking and administration	Questionnaire	Caregivers: assisting a loved one with medication management	Participants from a previous NHATS study, selected by a care intensity index. *N* = 1369 Caregiver age: > 18
O'Conor et al. (2021) USA	Qualitative	To characterize medication management by caregivers for older adults with multiple chronic conditions	Semi‐structured interviews	Caregivers: managing medication regimens for patients with ≥ 3 chronic conditions and medications	Participants from a previous study sample. *N* = 25 Caregiver mean age: 61 Caregiver gender: 17 females (68%) Care recipient mean age: 73
O'Conor et al. (2024) USA	Qualitative	To understand how people with MCI/dementia and family caregivers manage polypharmacy regimens and transitions in medication management responsibility	Semi‐structured interviews	Patients: MCI or dementia diagnosis, MoCA score 10–25, age > 60 years, ≥ 3 chronic conditions, ≥ 5 medications, English language, consent capacity, identified a caregiver Caregivers: age > 18 years—Primary caregiver for ≥ 6 months—English language	Dyads recruited from primary care, geriatric and memory disorder clinics in Chicago. *N* = 32 Caregiver mean age: 66 Caregiver gender: 23 females (71.9%). Care recipients mean age: 78
O'Quin et al. (2015) USA	Qualitative	To assess caregivers' and older adults' perceptions of medication management barriers and identify community solutions	Semi‐structured interviews	Patients: Age ≥ 60 years; taking medication daily for at least 10 consecutive days in the past 6 months Caregivers: assisting an elderly person meeting the above criteria	Recruitment via snowball sampling by stakeholders and key informants. *N* = 17 Caregiver mean age: 66 Caregiver gender: 15 females (88%) Care recipient mean age: 72
Onda et al. (2024) Japan	Quantitative, cross‐sectional	To clarify the sense of burden and factors influencing medication management among family caregivers of elderly patients with dementia	Online survey	Patients: chronic medication use caregivers: managing a family member with dementia at home	Preliminary screening on the Kanden CS Forum website. *N* = 100 Caregiver mean age: 55 Caregiver gender: 58.9% females Care recipient mean age: 84
Pereira et al. (2022) Switzerland	Qualitative	To identify and categorize stressors experienced and coping strategies adopted by older adults, informal caregivers and healthcare professionals in managing medications post‐hospital discharge	Semi‐structured interviews	Caregivers: age ≥ 18 years, identified as primary therapy manager by patient Patients: age ≥ 65 years, hospitalized within the past 90 days, taking ≥ 5 daily medications	Participants recruited by research nurses from a regional hospital and community health centre. *N* = 17 caregiver mean age: 68 (range: 48–86), care recipient mean age: 81
Polenick et al. (2020) USA	Quantitative, cross‐sectional	To examine caregiving stress factors and resources related to medication management, their association with caregiver role overload and whether these associations vary by patients' chronic conditions and dementia status	Questionnaire	Patients: enrolled in Medicare, age ≥ 65 years, living in contiguous US states, receiving unpaid caregiving assistance	Participants identified through NHATS and NSOC studies. *N* = 377 caregiver mean age: 72 caregiver gender: 188 females (50.09%)

Abbreviations: MCI, mild cognitive impairment; MoCA, Montreal Cognitive Assessment; NHATS, National Health and Aging Trends Study; NSOC, National Study of Caregiving.

Regarding geographic distribution, nine studies were conducted in the United States (LaValley et al. 2023; Li and Look 2022; Look and Stone 2018, 2019; Noureldin and Plake 2017; O'Conor et al. 2021; O'Conor et al. 2024; O'Quin et al. 2015; Polenick et al. 2020), two in the United Kingdom (Lawson et al. 2022; Lim et al. 2024), one in Spain (Gil‐Hernández et al. 2024), one in Germany (Lampert et al. 2020), one in Japan (Onda et al. 2024), one in Switzerland (Pereira et al. 2022) and one in Australia (El‐Saifi et al. 2019).

Regarding populations, three studies focused on informal caregiver of older people with dementia (Lim et al. 2024; O'Conor et al. 2024; Onda et al. 2024), while the remaining studies included older adults without specific cognitive diagnoses. Sample sizes varied considerably, with the largest sample reported by Noureldin and Plake (2017), with 1369 participants, and the smallest sample by Lampert et al. (2020), which included 11 participants.

Regarding the study setting, six studies were conducted in home‐based care contexts (Gil‐Hernández et al. 2024; Lampert et al. 2020; Lim et al. 2024; Onda et al. 2024; Pereira et al. 2022; Polenick et al. 2020), while one study was conducted in a hospital setting (Bruce et al. 2020). The remaining studies did not explicitly report the study setting. However, all included studies focused on medication management performed by informal caregivers of community‐dwelling older adults.

### 3.2 Risk of Bias Assessment

Among the five cross‐sectional studies (Table 2), the risk of bias was moderate overall. Inclusion criteria, contextual details and statistical analyses were generally appropriate. However, one study lacked clear strategies to manage confounding factors (Onda et al. 2024), and in some studies, outcome measurement was not always evaluated as valid or reliable (Li and Look 2022; Noureldin and Plake 2017; Onda et al. 2024).

**TABLE 2:** JBI critical appraisal tool checklist for analytical cross‐sectional studies.

	Gil‐Hernández et al. (2024)	Li and Look (2022)	Noureldin and Plake (2017)	Onda et al. (2024)	Polenick et al. (2020)
Were the criteria for inclusion in the sample clearly defined?	+	+	+	−	+
Were the study subjects and the setting described in detail?	+	+	+	+	+
Was the exposure measured in a valid and reliable way?	N/A	N/A	N/A	N/A	+
Were objective, standard criteria used for measurement of the condition?	+	+	+	−	+
Were confounding factors identified?	+	+	+	−	+
Were strategies to deal with confounding factors stated?	+	+	+	−	+
Were the outcomes measured in a valid and reliable way?	+	−	−	−	+
Was appropriate statistical analysis used?	+	+	+	+	+

In the 11 qualitative studies (Table 3), the overall risk of bias was low. Methodological coherence and rigour were generally strong. However, four studies did not address researcher reflexivity (El‐Saifi et al. 2019; Lampert et al. 2020; O'Conor et al. 2021; O'Conor et al. 2024).

**TABLE 3:** JBI critical appraisal tool checklist for qualitative studies.

	El‐Saifi et al. (2018)	Lampert et al. (2020)	LaValley et al. (2023)	Lawson et al. (2022)	Lim et al. (2024)	Look and Stone (2019)	Look and Stone (2019)	O'Conor et al. (2021)	O'Conor et al. (2024)	O'Quin et al. (2015)	Pereira et al. (2022)
Is there congruity between the stated philosophical perspective and the research methodology?	+	−	+	+	+	+	+	+	+	+	−
Is there congruity between the research methodology and the research question or objectives?	+	+	+	+	+	+	+	+	+	+	+
Is there congruity between the research methodology and the methods used to collect data?	+	+	+	+	+	+	+	+	+	+	+
Is there congruity between the research methodology and the representation and analysis of data?	+	+	+	+	+	+	+	+	+	+	+
Is there congruity between the research methodology and the interpretation of results?	+	+	+	+	+	+	+	+	+	+	+
Is there a statement locating the researcher culturally or theoretically?	N/A	N/A	N/A	N/A	N/A	N/A	N/A	N/A	N/A	N/A	N/A
Is the influence of the researcher on the research, and vice versa, addressed?	−	−	+	+	+	+	+	−	−	+	+
Are participants, and their voices, adequately represented?	+	+	+	+	+	+	+	+	+	+	+
Is the research ethical according to current criteria or, for recent studies, and is there evidence of ethical approval by an appropriate body?	+	+	+	+	+	+	+	+	+	+	+
Do the conclusions drawn in the research report flow from the analysis, or interpretation, of the data?	+	+	+	+	+	+	+	+	+	+	+

### 3.3 Synthesis of Results

**FIGURE 2:**
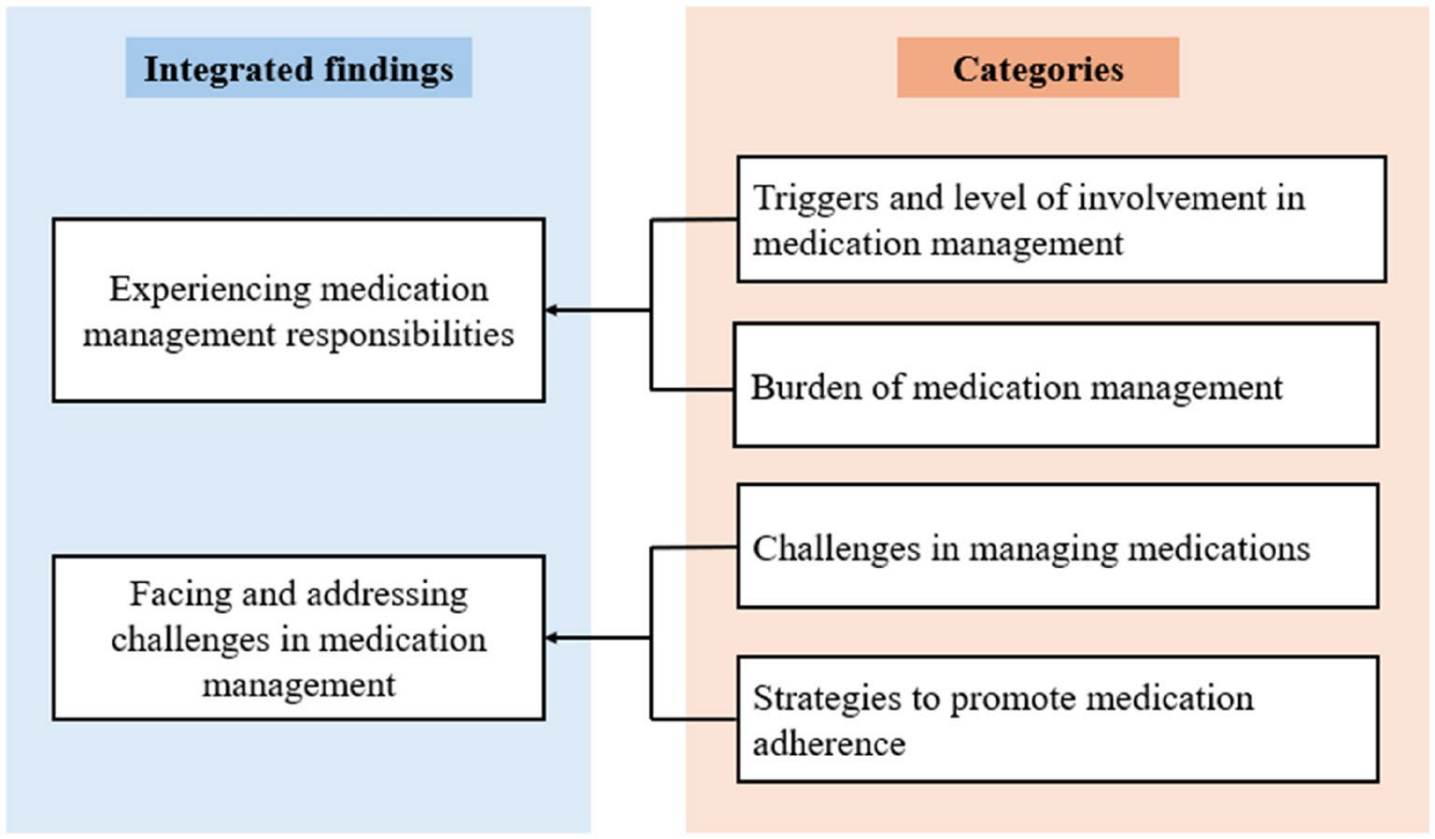
Categories and integrated findings.

Two integrated findings emerged from the analysis: ‘Perceiving and enact medication management responsibilities’ and ‘Facing and addressing challenges in medication management’ (Figure 2).

#### 3.3.1 Perceiving and Enact Medication Management Responsibilities

This integrated finding, including two categories called ‘Triggers and level of involvement in medication management’ and ‘Burden of medication management’, explores caregivers' level of involvement in medication management and the factors that influence their perceived burden associated with medication management.

##### 3.3.1.1 Triggers and Level of Involvement in Medication Management

Across the included studies, caregivers consistently reported that their involvement in medication‐related tasks increased as patients became more dependent in their activities of daily living (ADLs) (Look and Stone 2019; O'Conor et al. 2021; O'Conor et al. 2024). Changes in the patient's physical and/or cognitive health were frequently cited as the main triggers for caregivers to take over medication responsibilities (Look and Stone 2019), often following acute health events:

The third [heart attack] happened two days after the second… because he wasn't taking his medication. That's when I stepped in. (Lawson et al. 2022)

In the case of patients with dementia, the transition to caregiver‐led medication management often occurred when signs of cognitive decline, such as memory lapses, inattention or executive dysfunction, became evident, or when medication intake errors were observed (O'Conor et al. 2024).

We recently had to change [my levothyroxine], it was my responsibility since it needed to be taken so early. When I got up, he would say, ‘Did you take your pill?’ I'm like, ‘Yes, I think so.’ Coming to find out, last week my thyroid level was 20.45, which was way out of whack so then he counted the pills, went back to when we got them, and that's when he [started helping]. (O'Conor et al. 2024).

As regarding the level of involvement, caregivers who were fully involved reported managing all aspects of the medication process, including obtaining medications, preparing pill organizers, administering doses and monitoring for adverse effects (Look and Stone 2019; O'Conor et al. 2021). Several caregivers explicitly stated that they administered medications themselves (Li and Look 2022; Look and Stone 2018, 2019; Noureldin and Plake 2017; O'Conor et al. 2021):

I help her with the nebuliser because she used to be very good at it, but with dementia now, she's… you know… (Look and Stone 2018)

In one study, 8.7% of caregivers administered injectable medications, out of a total sample of 1813 participants (Noureldin and Plake 2017), and another reported a slightly higher proportion of 12.4% among 377 caregivers (Polenick et al. 2020).

Another commonly reported task was obtaining medications (Lawson et al. 2022; Li and Look 2022; Look and Stone 2018; Noureldin and Plake 2017; Polenick et al. 2020). Specifically, one study involving 1813 caregivers found that 55.9% helped their relative order medications (Noureldin and Plake 2017), and another study involving 377 spousal caregivers reported a higher proportion of 64.9% (Polenick et al. 2020). Some caregivers used online platforms for prescription refills:

The system went digital and she doesn't know how to use computers… I know how to order online, so I do it. (Lawson et al. 2022)

Regarding factors impacting the level of involvement in medication management, co‐residing with the care recipient emerged as strongly associated (Look and Stone 2019). In one study involving 100 caregivers, 67.8% lived with the patient (Onda et al. 2024). Co‐residing caregivers were generally responsible for the entire medication process, from ordering medications to direct administration (Look and Stone 2019). In contrast, non‐co‐residing caregivers displayed variable levels of involvement; in some cases, they remained deeply engaged, while in others, their support was limited to specific tasks:

I have to keep track of how many pills are actually in that bottle because she's still in her apartment, she's independent, and she fills her own pillbox. But I need to keep an eye on how fast we're going through them—like eye drops and other things—you know, that run out too quickly. (Look and Stone 2019)

##### 3.3.1.2 Burden of Medication Management

Several caregivers perceived medication management as a distinct responsibility, separate from other caregiving duties (El‐Saifi et al. 2019; Lawson et al. 2022). Emotional responses to this duty vary significantly, ranging from acceptance to feeling of being overwhelmed (O'Conor et al. 2024):

I feel constantly overwhelmed. Especially for someone who is not a nurse or a doctor—I feel like I need medication myself. I've experienced all the physical symptoms of being sick because of the stress of managing meds… (O'Conor et al. 2024)

In a study on 100 caregivers, almost 40% reported experiencing burden specifically related to medication management (Onda et al. 2024). In addition, in another study, caregivers described the emotional burden associated with being held responsible when medication adherence was compromised (El‐Saifi et al. 2019):

You know they need to take it, and you worry, … something might happen. And it'll be your fault. (El‐Saifi et al. 2019)

Individual caregiver characteristics were also found to influence the level of caregiver burden related to medication management, with greater distress reported among women, younger caregivers, and those with one or more chronic health conditions (Polenick et al. 2020). Further evidence indicated increased burden among caregivers responsible for administering injectable medications or caring for individuals with five or more chronic conditions (Polenick et al. 2020).

Other key contributors to this burden included complex medication regimens, a high number of daily doses, care recipients' refusal to take medications, poor compatibility with daily routines, and swallowing difficulties (Onda et al. 2024).

Contextual caregiving factors, such as providing care to individuals with dementia, performing nursing‐related tasks (i.e., tasks that extend beyond basic caregiving and are typically undertaken within the scope of healthcare practice, such as medication administration, monitoring for side effects, and treatment management), having frequent interactions with healthcare professionals, and supporting activities of daily living, also contributed to increased burden related to medication management (Polenick et al. 2020).

#### 3.3.2 Facing and Addressing Challenges in Medication Management

This integrated finding, including two categories ‘Challenges in managing medications’ and ‘Strategies to promote medication adherence’, explores the various challenges caregivers face when managing medications, as well as the strategies they adopt to promote adherence.

##### 3.3.2.1 Challenges in Managing Medications

Managing complex medication regimens for older adults presented numerous challenges for caregivers. Polypharmacy complicated the process, making it difficult to maintain adherence and recall the names and purposes of medications (O'Quin et al. 2015), that is further exacerbated when caregivers had to administer various formulations, such as tablets, liquids, insulin pens, inhalers, infusions and eye drops, as well as challenges related to packaging (Lampert et al. 2020):

The blister is too large, and the air inside prevents you from getting the pills out. (Lampert et al. 2020)

Dose preparation was another recurring issue, particularly when pills had to be split or crushed:

Sometimes it's hard to split the pills. (Lampert et al. 2020)

In addition, the use of weekly pill organizers, although commonly used and helpful, was also identified as time‐consuming and prone to errors (Lim et al. 2024):

Preparing the pills in advance is useful, but it takes a lot of time and can be confusing. (Lim et al. 2024)

Other challenges are related to the patient's characteristics. One commonly reported was the care recipient's resistance to assistance, with some refusing help or insisting on managing their own medications independently (Look and Stone 2018; O'Conor et al. 2021; Onda et al. 2024):

No, she said. I know when to take my pills. I know. (O'Conor et al. 2021)

In a study involving 100 caregivers, 56.7% reported difficulties in managing medications, of which 49% were attributed to the patient's forgetfulness, and 31.4% to swallowing difficulties, often exacerbated by cognitive decline (Onda et al. 2024). Patient's cognitive symptoms not only delayed medication intake but also contributed to missed prescription renewals (Lim et al. 2024). In addition, caregivers also acknowledged the potential risks of easy access to medications for individuals with dementia (Lim et al. 2024). Additional barriers included strained family relationships, lack of illness awareness, a strong desire for autonomy of the older adult, and concerns over privacy (O'Conor et al. 2024).

In addition to patient‐related challenges, caregivers also faced systemic and informational barriers that further complicated medication management. Many caregivers highlighted a lack of accessible information about medications (El‐Saifi et al. 2019; Look and Stone 2018; O'Quin et al. 2015), which undermined their confidence in safely managing drug regimens. To fill these gaps, some turned to online resources:

I check different sites… not just one. (Lawson et al. 2022)

Honestly, I rely on Google… to look up medications and ask questions. (El‐Saifi et al. 2019)

Other reported barriers included the cost of medications, especially when insurance coverage was incomplete (O'Quin et al. 2015), and concerns about side effects:

It scares me… those side effects… I don't want to misinterpret something and put my dad at risk. (Lawson et al. 2022)

Caregivers also expressed concern about the lack of coordination between specialists and primary care providers regarding medications, especially after hospitalization (O'Quin et al. 2015):

There's no real coordination between specialists and the primary doctor… and then you add a hospitalisation… things change. (O'Quin et al. 2015)

Numerous caregivers reported difficulties interacting with healthcare professionals, often due to staff shortages, high turnover, or a general lack of recognition of the caregiver role (Lawson et al. 2022; Pereira et al. 2022):

Every caregiver should receive that kind of respect… It's a huge responsibility to manage someone else's medications. (Lawson et al. 2022)

They just assume you can do it. They don't realise that maybe you can't. (Lawson et al. 2022)

The complexity of managing medications often led to errors. In one study involving 685 caregivers, 57% reported having made at least one mistake, including dosage errors (44%), mixing up medications (20%), mishandling medications (31%), and incorrect storage (15%) (Gil‐Hernández et al. 2024).

Key barriers to medication adherence included non‐cohabitation, which required caregivers to reorganize medication routines based on their own availability rather than the patient's preferences (Look and Stone 2019; O'Conor et al. 2024). Social isolation and the lack of support networks, particularly in rural areas, were also perceived by caregivers as significant obstacles to adherence (Look and Stone 2019).

She lives by herself. I live close, but not with her. Her cognitive abilities and the decline that's happening there, I think, makes it very difficult for me to know what happens. If I get there and there's still pills in the box, I don't know if she thought she had to refill them or if she actually didn't take them. (O'Conor et al. 2024)

##### 3.3.2.2 Strategies to Manage Medications and Support Adherence

Despite many difficulties, caregivers adopted several strategies to manage medications, including supporting adherence, developing routines and preserving patient autonomy (El‐Saifi et al. 2019; Lampert et al. 2020; LaValley et al. 2023; Lim et al. 2024; Look and Stone 2018). Among the most commonly used tools, there were pill organizers (blister packs), which proved particularly useful when multiple caregivers were involved in the medication routine (LaValley et al. 2023; O'Quin et al. 2015):

… If I'm not home and my husband is with a helper, she can give him his pills if needed. (El‐Saifi et al. 2019)

Caregivers often developed personalized systems to monitor adherence. These included creative adaptations of household items:

It's our modified pill container—just glass cups. When he takes his meds, he turns the cup upside down. (LaValley et al. 2023)

Reminder systems played a key role, ranging from alarms on patients' phones to traditional timekeeping methods (El‐Saifi et al. 2019; Look and Stone 2018):

We set two alarms, one in the morning and one in the evening, so she remembers to take her pills. (Look and Stone 2018)

Monitoring techniques varied widely. In one study, caregivers reported checking ingestion directly (59%), inspecting medication containers (31%), asking the care recipient (7%), or using other strategies (3%) (Onda et al. 2024). Some utilized smartphones and tablets equipped with reminder applications, and caregivers expressed interest in a customized, purpose‐built app (El‐Saifi et al. 2019):

*[the medication app]* It should be built from scratch. (El‐Saifi et al. 2019)

Smartphones were also used to keep medication lists readily available:

… I always have it with me… It's right here, in my phone notes. (Look and Stone 2018)

However, not all caregivers embraced digital tools. Some voiced a preference for simplicity and interpersonal connection over technological solutions (LaValley et al. 2023):

*It's the human connection, not technology, that's key in caregiving*. (LaValley et al. 2023)

Manual tracking methods, such as handwritten lists, remained common and were often used to support medication schedules (El‐Saifi et al. 2019; LaValley et al. 2023):

I write a list. I even have copies. (LaValley et al. 2023)

Establishing consistent routines was frequently cited as a successful strategy for integrating medications into daily life (Lawson et al. 2022; Lim et al. 2024; Look and Stone 2018). In one study, 84% of caregivers believed that medication routines had become a natural part of the patient's lifestyle (Onda et al. 2024). Some caregivers placed medications in visible locations to support adherence (Look and Stone 2018):

So, I put the nighttime pills by the microwave and the morning pills where he eats breakfast, but he still gets confused sometimes. (Look and Stone 2018)

Strategies to minimize medication errors included receiving targeted training, becoming more familiar with the pharmacological regimen, and providing more intensive or full‐time care (Gil‐Hernández et al. 2024).

Despite differing levels of involvement, many caregivers emphasized the importance of preserving the patient's autonomy and independence whenever possible:

I try to maintain her independence as much as possible, but I still monitor everything. (O'Conor et al. 2024)

He wants to be independent. He doesn't like me telling him what to do. (Look and Stone 2019)

## 4 Discussion

The purpose of this mixed method systematic review was to synthesize evidence on the experiences of informal caregivers in managing medications of older adults. The review identified two main dimensions of these experiences: ‘Perceiving and enact medication management responsibilities’ and ‘Facing and addressing challenges in medication management’. Overall, the included studies were of acceptable methodological quality, with qualitative designs showing strong rigour and quantitative studies presenting some limitations. Taken together, the integrated findings of this review can be considered sufficiently reliable.

Our first integrated finding highlights the dynamic nature of caregiver involvement, which typically intensifies as care recipients experience physical or cognitive decline, acute health events such as hospital discharge, episodes of major illness or observable non‐adherence.

Several studies included in this review reported that caregivers were responsible for multiple stages of the medication process, including prescription, organizing pillboxes, administering medication and monitoring for adverse effects. This finding is consistent with previous studies demonstrating that medication management is among the most frequent and burdensome tasks undertaken by informal caregivers (Alkhaldi et al. 2025; Reinhard et al. 2012).

In our review, co‐residency emerged as a key determinant of the intensity and scope of caregiver involvement. Caregivers who lived with the older adult typically took on end‐to‐end medication responsibilities, while non‐resident caregivers were more likely to adopt supervisory or supporting roles. This observation is consistent with Alkhaldi et al. (2025), who also found that the degree of caregiver involvement varied significantly and was strongly influenced by the level of independence and living arrangements of the care recipient.

This review found that caregiver burden varied across populations and was influenced by the complexity of the medication regimen and the nature of administration tasks, particularly when parenteral therapies such as injectable medications were involved. This is consistent with the results of Alkhaldi et al. (2025), who conceptualized caregiver responsibilities across two interrelated domains: physical tasks (e.g., prescription management, medication preparation) and cognitive tasks, such as decision‐making, adherence evaluation through routine monitoring strategies embedded in daily life, such as placing notes or medications in visible places or linking medication intake to daily activities to verify whether doses had been taken as prescribed (Alkhaldi et al. 2025). The interplay between these dimensions often places caregivers under sustained pressure, especially in the absence of adequate support systems (Alkhaldi et al. 2025).

The other integrated finding of this review focuses on the range of challenges caregivers face in supporting adherence to medications and the strategies they adopt in response. Common difficulties included navigating complex therapeutic regimens, administering multiple formulations (e.g., pills, inhalers, eye drops) and managing resistance from care recipients, particularly among individuals with cognitive impairment or dementia. This findings are consistent with previous reviews (Alkhaldi et al. 2025; Aston et al. 2017; Gillespie et al. 2014), which identified similar challenges related to medication refusal, lack of caregiver training and the increased complexity of managing pharmacotherapy in home settings. For example, Alkhaldi et al. (2025) reported difficulties related to distinguishing between similar medications, ensuring safe storage at home and administering treatments such as inhalers, factors also identified in the present review. In addition, several of the studies included in the present review directly addressed medication management in the context of dementia care, highlighting how cognitive deterioration associated with the condition adds an additional layer of complexity to caregiving, particularly in relation to medication adherence and administration. This issue is aligned with the results of a previous systematic review by Aston et al. (2017), which specifically examined how informal caregivers manage pharmacological therapies for individuals with dementia. Their findings reinforce the notion that medication management in this population is a particularly demanding task, frequently associated with increased caregiver burden.

In addition to practical challenges, this review identified systemic barriers that further complicate caregiving, including limited access to accurate medication information, poor communication with healthcare professionals, lack of integration between providers and limited recognition of the caregiver role. These findings align with previous studies, which have also documented poor communication, fragmentation of care and insufficient recognition of informal caregivers (Aston et al. 2017) F. A. Davis Company. In particular, the lack of recognition is often reinforced by implicit expectations that caregivers should manage complex medication‐related tasks without formal training (Lawson et al. 2022; Pereira et al. 2022).

In our review, caregivers also expressed concern about potential side effects and the fear of making mistakes, feelings often exacerbated by the absence of clear guidance and the reliance on unverified online sources, as reported by another review (Alkhaldi et al. 2025). In this review, medication errors were reported in one included study, highlighting a potentially underexplored issue and the need to develop targeted interventions and further explore this issue in future research. Caregivers also expressed concern about potential side effects and fear of making mistakes, particularly in the absence of clear guidance and when relying on unverified online sources. These findings are supported by previous evidence, which suggests that targeted interventions can enhance caregivers' medication knowledge and self‐efficacy (Wagle et al. 2018). However, the review also highlighted the lack of robust evidence on the impact of such interventions on clinical outcomes and healthcare utilization.

Despite these barriers, caregivers often demonstrate creativity and adaptability in promoting adherence. Common strategies include the use of weekly pill organizers, alarms, handwritten medication lists and individualized routines, according to another recent review (Alkhaldi et al. 2025). Although widely adopted, in our review such tools typically emerge in the absence of formal instruction or professional support. This reflects a larger gap in caregiver education and recognition, which contributes to anxiety, medication errors and reduced confidence in the management of therapies (Rodziewicz et al. 2024). The role of digital tools remains mixed, with some caregivers reporting benefits from smartphone apps and reminders and others preferring simpler, low‐tech solutions that preserve interpersonal interaction (LaValley et al. 2023).

Our findings indicate that the challenges reported by caregivers, such as managing complex medication regimens, navigating healthcare systems and balancing patient autonomy with safety, appear consistent across different contexts, suggesting that several of the identified experiences may be transferable to other settings. However, most of the included studies were conducted in high‐income countries, which may limit the applicability of the findings to healthcare systems with different organizational structures and caregiver support services. In high‐income contexts, caregivers may have greater access to technological tools and healthcare resources that can support medication management and adherence. Furthermore, family structures in many high‐income countries are often characterized by older adults living alone (Djundeva et al. 2019), which may influence the extent and nature of caregiver involvement in medication management. These contextual factors may differ substantially in low‐ and middle‐income countries, where informal caregiving arrangements, availability of healthcare services, and access to technological support may vary.

This review presents some limitations. The review included only studies published in English, which may have led to the exclusion of relevant research conducted in other languages and contexts. Furthermore, the last 10 years were considered, potentially excluding relevant studies. However, this choice was made to ensure updated results, considering the rapid evolution of medications available.

The findings of this review highlight the need to develop structured support interventions for informal caregivers involved in the management of medications for older adults. Caregivers consistently reported the need for clear, practical information about pharmacological therapies, as well as personalized education to build confidence and reduce errors in the home setting. Addressing these needs requires a deeper understanding of caregivers' real‐life experiences, informational gaps and emotional responses related to medication management.

Nurses and other healthcare professionals play a key role in identifying caregiver concerns, providing anticipatory guidance and embedding educational support within discharge planning and ongoing community care. In this context, strengthening the preparation of healthcare professionals, in particular nurses, in supporting informal caregivers is essential (Gil‐Hernández et al. 2024).

Future research should be focused on further understanding the medication management errors and related causes, to develop and evaluate co‐design interventions that promote medication adherence while alleviating caregiver burden. At the policy level, greater recognition of informal caregivers as essential partners in care is needed, alongside the development of integrated support systems that improve access to reliable medication information and enhance coordination across healthcare settings.

Finally, these findings also have implications for environmental sustainability and planetary health. Inadequate medication management may contribute to medication waste, inappropriate pharmaceutical use, and avoidable healthcare utilization, thereby increasing the environmental footprint of healthcare systems (Ravinetto et al. 2025). Supporting caregivers in safe and efficient medication practices may therefore contribute not only to improved patient and caregiver outcomes, but also to a more sustainable use of healthcare resources.

## 5 Conclusion

This review highlights how informal caregivers often assume responsibility for medication management of older adults, a role that, while essential, can be a significant burden. Nowadays, caregivers navigate complex medication regimens, often relying on self‐developed tools and strategies to manage adherence and administration at home, which might result in mistakes.

A notable gap persists between the expectations placed on caregivers and the level of training, information and professional support they receive. Many caregivers report feeling unprepared and unsupported in managing such a critical aspect of care. These findings underscore the need for structured ongoing interventions led by healthcare professionals that offer education, guidance and emotional support, beginning with the initial transition to responsibility and continuing throughout the caregiving journey. Tailored support strategies that account for the individual needs of caregivers, the technological capacity, and the dynamics of the relationship are essential. Such interventions not only have the potential to improve medication adherence and patient safety but also to reduce caregiver burden.

## Author Contributions

**Daniela Avgustini:** conceptualization, methodology, formal analysis, writing – original draft. **Mayra Veronese:** data curation, investigation, writing – review and editing. **Anna Brugnolli:** supervision, writing – review and editing. **Matteo Danielis:** supervision, writing – review and editing. **Jessica Longhini:** conceptualization, methodology, formal analysis, writing – original draft.

## Funding

The authors have nothing to report.

## Conflicts of Interest

The authors declare no conflicts of interest.

## Supporting information


**File S1:** Preferred Reporting Items for Systematic Reviews and Meta‐Analyses Checklist.
**File S2:** The search strategies.

## Data Availability

The data that support the findings of this study are available from the corresponding author upon reasonable request.
